# A Pilot Study of an Endoscope-Assisted Minimally Invasive Cortical Access System (MICAS) for Chronic Subdural Hematoma Evacuation

**DOI:** 10.7759/cureus.101367

**Published:** 2026-01-12

**Authors:** Sandhya R Palit, Hirotaka Hasegawa, Yuki Shinya, Stephen T Kuehn, Mark A Benscoter, Gregory A Worrell, Sanjeet Grewal, Jamie J Van Gompel

**Affiliations:** 1 Department of Neurologic Surgery, Mayo Clinic, Rochester, USA; 2 Department of Neurosurgery, Saitama Medical Center, Saitama Medical University, Saitama, JPN; 3 Department of Neurosurgery, University of Tokyo, Tokyo, JPN; 4 Department of Engineering, Mayo Clinic, Rochester, USA; 5 Department of Biomedical Engineering, Mayo Clinic, Rochester, USA; 6 Department of Neurology, Mayo Clinic, Rochester, USA; 7 Department of Neurosurgery, Mayo Clinic, Jacksonville, USA

**Keywords:** chronic subdural hematoma, cortical access system, endoscopic hematoma evacuation, minimally invasive, minimally invasive approach, prospective study

## Abstract

Objectives: Chronic subdural hematoma (CSDH) is a common neurosurgical condition, particularly in the elderly. While traditional surgical options such as craniotomy and burr hole craniostomy remain prevalent, endoscopic evacuation may offer a minimally invasive approach. This study aimed to evaluate the safety and feasibility of a novel device, the Minimally Invasive Cortical Access System (MICAS), with flexible endoscopic evacuation of CSDH.

Methods: A prospective study was conducted from April 2021 to April 2022, enrolling five patients with symptomatic CSDH. Patients underwent endoscopic hematoma evacuation using the MICAS device through a single burr hole. Surgical outcomes and device safety were assessed.

Results: The MICAS device was successfully used in all five patients, providing stability and maneuverability for the flexible endoscope. A single burr hole of average size 18.2 ± 6.2 mm was used. In four out of five cases, only one burr hole was used, reducing the need for additional incisions. Subdural drains were placed in four patients. Three patients proceeded to have middle meningeal artery embolization (MMAE). The procedure was well tolerated, without notable direct complications.

Conclusion: The MICAS device aided in endoscopic evacuation of CSDH, providing safe and effective protection of the cerebral cortex during the flexible endoscopic evacuation. Further studies with a larger sample size are warranted to validate these findings and explore the broader applications of this innovative device.

## Introduction

Chronic subdural hematoma (CSDH) is a relatively common condition, particularly in the elderly population, with an estimated annual incidence ranging from 1.7 to 20.6 cases per 100,000 people [1,2]. This trend is primarily driven by the aging demographic and the widespread use of anticoagulants and antiplatelet medications. CSDH has a gradual onset and is characterized by a slower progression of symptoms. Although CSDH may resolve spontaneously, an untreated hematoma can result in cerebral compression and/or a deteriorating neurological condition. The recurrence rate of CSDH, ranging from 12% following the initial surgical treatment, remains a significant concern [3]. While the introduction of adjunctive approaches, such as middle meningeal artery embolization (MMAE), has expanded treatment strategies, the overall reoperation rate remains relatively high, estimated at around 10-20% globally [3].

Endoscopic surgery has recently garnered attention as a treatment option for subdural hematomas (SDHs) due to its improved visualization and minimally invasive nature [4]. While neuroendoscopy has been used for many years, its application to SDHs has remained limited due to challenges in endoscope control and brain protection [5]. To address these issues, we developed a support device called the Minimally Invasive Cortical Access System (MICAS) that can be deployed into the burr hole. MICAS protects the brain at the entry point of the endoscope and improves its maneuverability within the subdural space. By using a flexible endoscope and reducing the number of burr holes, MICAS could potentially decrease the risk of brain injury associated with endoscopy maneuvers. In this study protocol, we aimed to evaluate the safety and feasibility of MICAS in reducing the damage to the cortical surface while performing endoscopic evacuation of CSDH.

## Materials and methods

Investigational device

**Figure 1:**
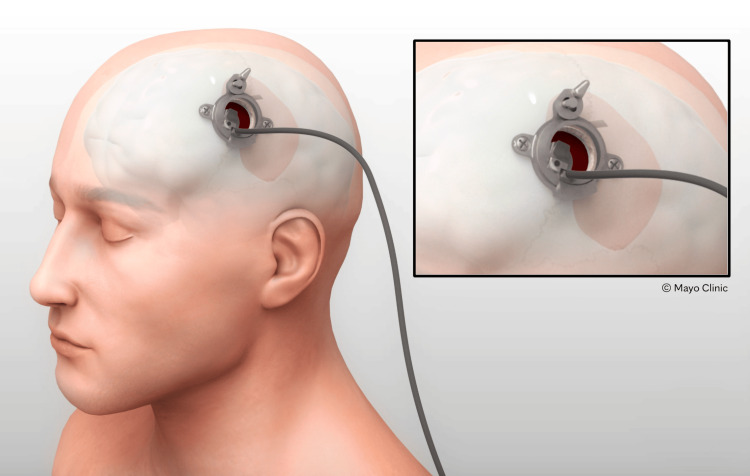
The Minimally Invasive Cortical Access System (MICAS) device intended for use Credit: Used with permission of Mayo Foundation for Medical Education and Research. All rights reserved.

**Figure 2:**
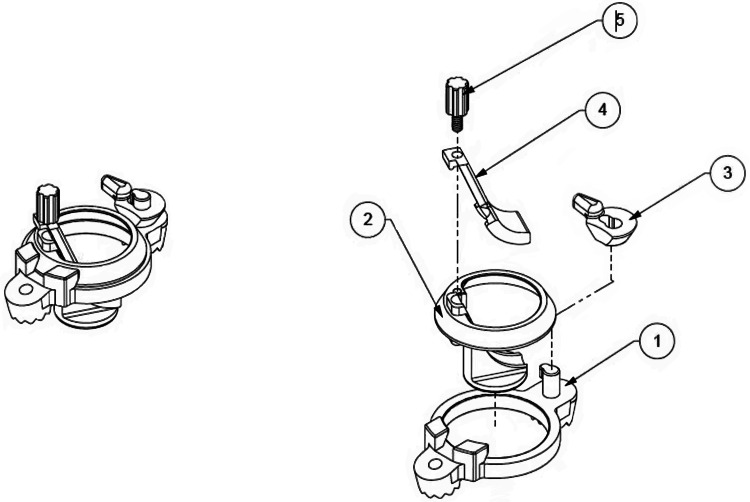
Minimally Invasive Cortical Access System (MICAS) device assembly and exploded view The MICAS features a metal base that attaches securely to the skull using a single screw (labeled 1, 2, and 3), providing a stable foundation for the device. Additionally, a mechanism (labeled 2, 4, and 5) is in place to firmly secure the endoscope, ensuring that its position remains stable and allowing safe use of its internal channels. Credit: Used with permission of Mayo Foundation for Medical Education and Research. All rights reserved.

The MICAS is a temporary stainless steel surgical tool that provides access to the subdural space through the cranium. The device is intended to protect the brain at the burr hole site during endoscopic subdural evacuation of CSDH, as well as potentially provide dead space if the brain is opposed to the dura at the entry area (Figure 1). The device is classified as a category B, Class II medical device and comprises five parts which can be assembled without special tools (Figure 2). The MICAS device was designed for the Karl Storz video Neuroendoscope (0-degree, 1.2 mm working channel, 35 mm working length, 2.8 mm outer diameter; Karl Storz SE & Co. KG, Tuttlingen, Germany). This device received FDA approval through an Investigational Device Exemption (IDE) application, allowing its use for this study.

MICAS aims to aid in control of the distal tip of the flexible endoscope by acting like a periscope and directing it parallel to the brain surface to enter the subdural space tangential to the cortex, allowing a 360° maneuverability. Simultaneously, it protects the brain at the burr hole site while working with the endoscope. The MICAS features a metal base that attaches securely to the skull using a single screw (labeled 1, 2, and 3 in Figure 2), providing a stable foundation for the device. Additionally, a mechanism (labeled 2, 4, and 5 in Figure 2) is in place to firmly secure the endoscope, ensuring its position remains stable and allowing for safe use of its internal channels.

**Figure 3:**
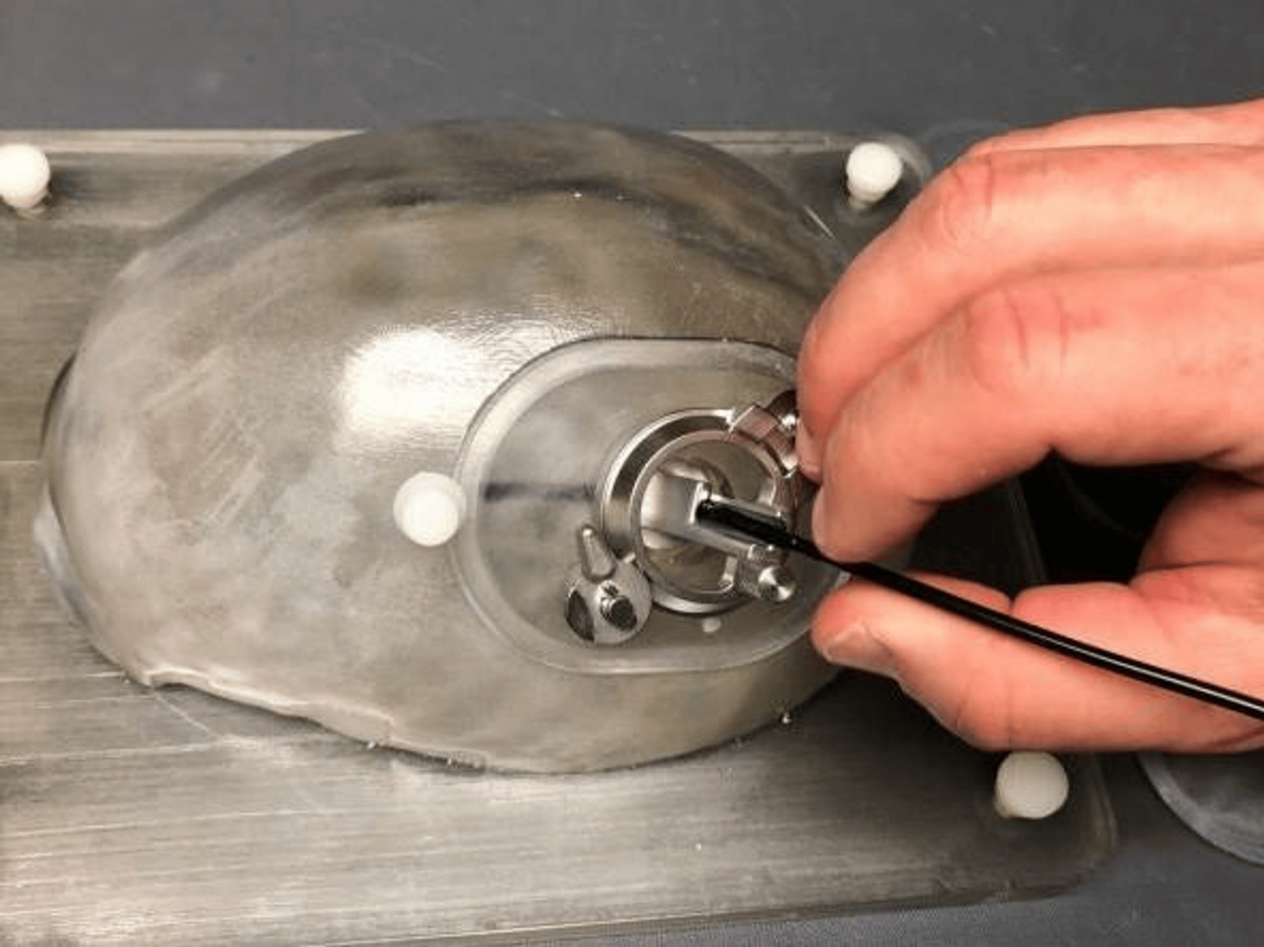
Deployment of the neuroendoscope in a benchtop model Credit: Used with permission of Mayo Foundation for Medical Education and Research. All rights reserved.

**Figure 4:**
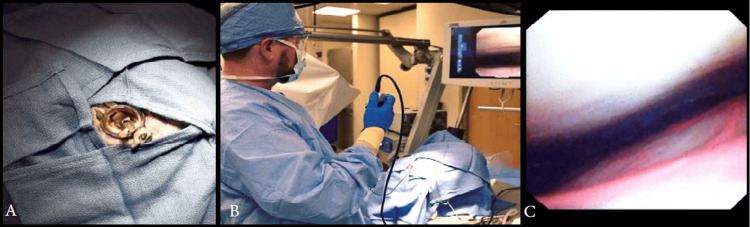
(A) Implanted Minimally Invasive Cortical Access System (MICAS). (B) Neuroendoscope manipulation with 360° rotation through the MICAS. (C) Neuroendoscopic view of the dura (top) and pia-encapsulated brain surface (bottom). Credit: Used with permission of Mayo Foundation for Medical Education and Research. All rights reserved.

Due to the lack of a suitable large animal model for studying SDH, the MICAS device was not tested on animals. Instead, the device was rigorously tested using a benchtop skull model to ensure it met all design requirements and performed as intended (Figure 3). Additionally, human cadaver studies were conducted to validate the device's effectiveness in real-world conditions and confirm that it met the needs of both users and stakeholders (Figure 4).

Study design

This prospective study from a single institution recorded patients from April 2021 to April 2022.

Sample size and study population

Our study included five patients aged 55 or older with symptomatic CSDH measuring at least 1 cm in thickness and requiring surgical intervention. Patients who declined to participate, were considered vulnerable, required chronic anticoagulation, were pregnant, had a history of SDH surgery, or had a Glasgow Coma Scale score below 8 were excluded from the study [6]. All patients in the study underwent endoscopic evacuation of their SDH using the MICAS device under general anesthesia.

Ethics statement

The study was conducted in accordance with the Declaration of Helsinki and was approved by the Mayo Clinic Institutional Review Board (IRB number 24-004217). Informed consent for study participation and de-identified publication of results was obtained from all participants prior to their enrollment. All patient data was collected and analyzed anonymously to ensure patient confidentiality.

Pre-operative preparation

Pre-operative computed tomography (CT) and magnetic resonance imaging (MRI) were performed in all cases. Cleaning, packaging, and sterilization of the MICAS device were performed within the neurosurgery core facilities at Mayo Clinic Hospital.

Surgical procedure

**Figure 5:**
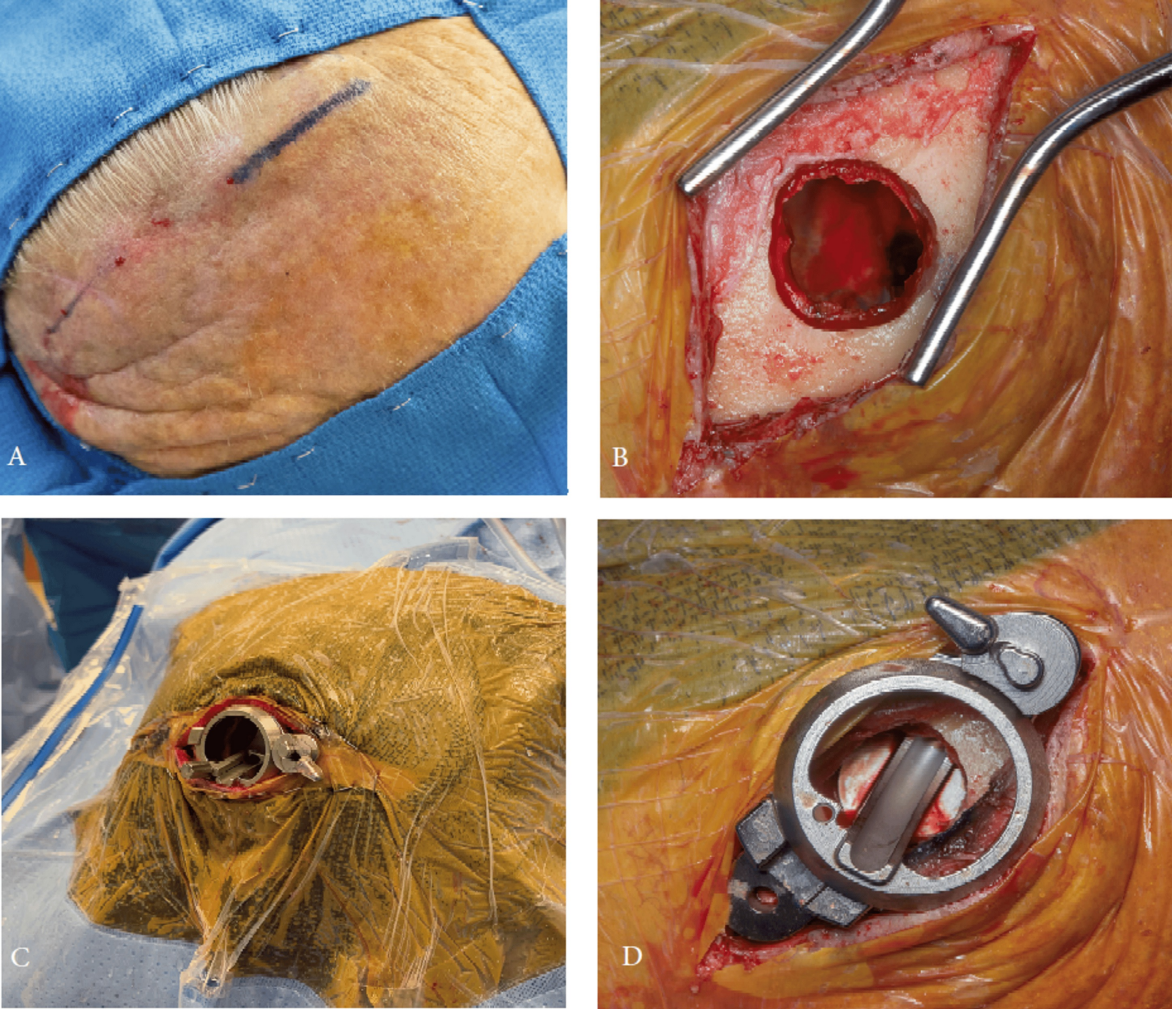
(A) Incision length of approximately 6 cm. (B) Burr hole measuring 12 mm to accommodate the MICAS. (C) Screw fixation of the MICAS into the burr hole with sterile draping. (D) Securely attached MICAS footplate showing the internal channel for passage of the flexible endoscope. Credit: Used with permission of Mayo Foundation for Medical Education and Research. All rights reserved.

The patients were positioned supine on the operating table with their heads resting on a donut pillow. The surgical sites were prepped and draped in a sterile fashion. A 5-6 cm linear skin incision was made over the central location of the SDH. The skin and soft tissues were dissected to expose the bone. A single burr hole of a maximum of 2.4 cm in diameter was drilled into the skull. The MICAS device, made of stainless steel, was assembled at the sterile back table. It was placed in the burr hole and secured with one small cortical screw (Figure 5). The dura was then excised. Initial hematoma evacuation was performed in the normal manner with irrigation of warmed normal saline through the burr hole. After satisfactory evacuation of the hematoma via return of clear irrigation fluid and re-expansion of the brain toward the skull, the endoscope was brought into the field and passed through the channel, and the subdural space was inspected. In areas of residual hematoma, irrigation was performed directly through the endoscope. At the discretion of the surgeon, a second burr hole was created to ensure appropriate evacuation, but the MICAS device was not used in the second burr hole. Once adequate evacuation was felt to have been achieved, the endoscope was withdrawn, and the MICAS was removed. The burr holes were closed with a single burr hole cover with gel foam underneath it, and the wounds were closed in anatomical layers using sutures or staples after extensive irrigation. A subdural drain was placed to facilitate drainage. All patients had a postoperative CT. Return of symptoms with re-accumulation or continued compression of the CSDH on the brain was considered a recurrence.

## Results

There were two male and three female patients aged 60 to 85 years with unilateral CSDH (Table 1). The etiology of the CSDH included both nontraumatic (n = 3) and traumatic (n = 2). The duration of CSDH varied, with two patients having symptoms for over four weeks (20 and seven weeks), while the others presented within one week of symptom onset. Preoperative imaging revealed significant midline shifts ranging from 6 mm to 13 mm. Septations were found in three patients on the initial imaging.

**Table 1:** Summary of patient data MMAE: middle meningeal artery embolization

Patient no.	Age/sex	Midline shift (mm)	Maximum diameter of hematoma (cm)	Burr holes size (mm)	Drain placed	Recurrence	Additional treatment
1	79/M	13	2.7	12.0	No	Yes	Reoperation and MMAE
2	60/F	6	1.6	22.2	Yes	No	None
3	85/F	7	10.9	24.6	Yes	No	Planned MMAE
4	75/M	13	1.9	21.0	Yes	No	Planned MMAE
5	70/F	10	2.1	11.0	Yes	No	None

All the patients underwent MICAS-assisted endoscopic subdural evacuation by a single surgeon. The burr hole to accommodate the custom MICAS endoscopic access frame (footplate) was made on the frontal area behind the hairline, depending on the side of the hematoma, regardless of the extension. Except for one, all patients had a single burr hole with an average size of 18.2 ± 6.2 mm (Table 1).

The endoscope visualized extensive membrane formation in both the anterior and posterior regions beyond the craniotomy site in two patients (Video 1). In one patient, a second burr hole was drilled on the parietal region after identifying persistent membranes and blood posteriorly via the flexible endoscope (Table 2). A subdural drain was placed in four patients, ensuring the tip was on the surface of the brain. The largest SDH, approximately 11 cm in diameter with septations, was successfully drained. No patients had new symptoms or neurological findings postoperatively. There were no intraoperative complications, such as infections with the device. CT demonstrated no new brain injury, nor did follow-up imaging.

**Video 1:** Use of the novel MICAS device for the evacuation of chronic subdural hematomas The device is introduced through a single burr hole and secured to the skull with two small screws and a locking mechanism, creating a protected channel for the flexible endoscope. Preoperative and postoperative imaging of the patients in the series is shown, with the colored area indicating a chronic subdural hematoma. The multi-directional capabilities of MICAS allow for comprehensive endoscopic assessment of the subdural space, irrigation, and suction. MICAS: Minimally Invasive Cortical Access System

**Table 2:** Surgical outcomes for patients (n = 5) MMAE: middle meningeal artery embolization

Variables	n (%)
Patients requiring two burr holes	1 (20)
Drain placement	4 (80)
Additional MMAE	3 (60)
Postoperative infection	0 (0)
Length of hospitalization, days (mean)	2-7 (3.9)
Readmission within 30 days	2 (40)
Postoperative death	0 (0)
Re-operation	1 (20)

Case 1: re-operation

As the first patient to be enrolled in the study had a cochlear implant (CI) over the parietal bone, attempting to avoid the device, we did not intend to place a second burr hole (Video 1). Due to the implant, it was decided not to use a monopolar cautery nor to have a subdural drain in place. After the hematoma evacuation, a flexible endoscope utilizing MICAS was employed to extend the visual field beyond the burr hole. This revealed a clot firmly adhered to the inferior aspect; however, to avoid making an incision near the CI, it was elected to close. He subsequently had persistent compression requiring a second surgery for the reopening of the prior left frontal burr hole, and creation of an additional left parietal burr hole superior to the CI for CSDH evacuation. This patient also went on to MMAE for the planned adjuvant treatment.

Four (80%) patients had drains placed to reduce the risk of hematoma recurrence after the first case. Except for one, all four patients with CSDH underwent planned adjuvant MMAE after the surgery with MICAS, which became standard in our practice over this time (Table 2) [7]. All the patients were successfully followed up on a long-term basis and reported no complications from the device. There were no deaths intra-operatively or during the recovery period.

## Discussion

Despite ongoing research, surgical treatment strategies for CSDH have remained relatively unchanged, though MMAE has recently been employed frequently [8]. The current surgical options widely practiced include twist drill craniostomy (TDC), involving small openings of less than 10 mm made using a twist drill, burr hole craniostomy (BHC), involving openings of 10-30 mm, and craniotomy, involving larger openings [1,7,9,10]. The Subdural Evacuating Port System (SEPS) is another temporary option for treating patients with chronic, subacute, and acute-on-chronic SDH at the bedside without needing to enter the intracranial space [10]. Our medical center has discontinued the use of minimally invasive TDC for treating CSDH due to a high rate of reoperations. Approximately one-third of patients who underwent this procedure required additional surgery [11].

MMAE is a promising new low-risk treatment option for CSDH and acute-on-chronic SDH. This minimally invasive procedure, performed by a multidisciplinary team including interventional radiologists, neurosurgeons, and interventional neurologists, works by stopping blood flow to the friable capillary nexus, leading to hematoma resolution [8]. It is noteworthy that MMAE gained significant traction during the time frame of this study, potentially contributing to the observed improvement in recurrence rates [12]. When performed in tandem, these two interventions can be more effective in treating CSDH because they are minimally invasive and can address both immediate and long-term problems [13].

Endoscopic evacuation is gaining popularity due to its minimal invasiveness and the ability to visualize and remove blood clots. Numerous studies have demonstrated the safety and effectiveness of endoscopic evacuation for SDH. However, one challenge with this technique is safely maneuvering the endoscope without causing damage to the cortex [14].

Rapid re-expansion of the brain that leads to trapping of the distally placed hematoma may obscure vision and make it difficult to advance the endoscope blindly. Yadav et al. [15] developed a modified retractor for use in endoscopic evacuation when there's limited space due to the brain swelling back up or a narrow hematoma cavity. This helps to gently retract the brain and its inner membrane, keeps the endoscope lens clean, and protects the cortex from damage. Made from a silicone tube that's cut and shaped to be easily inserted, with a suture added for easy removal, the modified retractor was reportedly used in five hematoma cavities with successful outcomes. The modified retractor was especially helpful because the doctors used rigid endoscopes and inserted a catheter to wash out the blood clot area. They found it difficult to move the rigid endoscope when the bony opening was not centered correctly [15]. The authors believe a device like MICAS, which allows for a flexible endoscope and a wider range of movement for evacuation, irrigation, and suction, would be particularly beneficial in these situations, as it is much like a standard stainless-steel retractor that they would use elsewhere to control the brain.

Use of a single versus a second burr hole to drain the CSDH remains a controversy among neurosurgeons. A meta-analysis comparing single and double BHC for treating CSDH found no significant differences in recurrence rates, complications, or mortality [16]. The MICAS device's 360-degree rotational capability enabled the endoscope to access the distal regions of the hematomas and visualize any residual blood clots, reducing the need for a second burr hole. Only one patient required two burr holes. The device effectively helped inspect the resection cavity for additional hematomas, membranes, and bleeding. It also provided visualization of the brain surface beyond the borders of a craniotomy as required during the evacuation of a CSDH. The need for further exploration via a second burr hole can be predicted after inspection with the flexible endoscope.

Nagasaka et al. [17] concluded that a balanced combination of irrigation, suction, and coagulation of the affected blood vessels with a multifunctional suction cannula resulted in reliable hemostasis in intracerebral hemorrhage [16]. Building upon this prototype, Yokosuka et al. [4] developed an irrigation suction cannula equipped with a malleable nozzle that could be readily inserted into the subdural space and maneuvered at various angles [4]. The MICAS device featured a distinct channel for passing the endoscope through (Video 1). The FDA-approved endoscope employed in this study incorporated an integrated irrigation and dry field suction system, thereby eliminating the need for multiple instruments.

Placing a subdural drain can help close the subdural space by bringing the outer and inner membranes closer together [18]. It is essential to make sure the drain tip is touching the brain's surface. The MICAS channel helps ensure the drain is placed parallel to the brain's surface, which can be checked with the endoscope before closing the incision. Though one patient did not have the subdural drain placed due to his CI, he subsequently developed a recurrence. Hence, remaining patients with SDH were closed with a drain in place.

Endoscopic surgeries for the brain have been performed for an extended period. Although most procedures necessitate a rigid endoscope to penetrate the cerebral cortex, certain surface-level interventions utilizing a flexible endoscope might benefit from the MICAS device. Patients with multiple areas of seizure onset may benefit from MICAS-assisted endoscopic grid placement targeting the seizure focus [19]. However, with the advent of frame-based, frameless stereotaxis and robotic-assisted techniques for stereoscopic electroencephalogram (sEEG) placement, there may be a diminishing need for BHC in neuromonitoring or neuromodulation.

Limitations

The MICAS was initially intended for use in a study involving 20 patients. However, due to supply chain disruptions during the pandemic, the distal chip became unavailable for the Storz flexible videoscope, leading to a significant reduction in device availability, as the device breaks frequently. Consequently, only five patients could be enrolled in the study before it was closed due to the inability to obtain the flexible endoscope from the company. The small sample size of five patients may limit the statistical power of the study. While the clinical trial is currently suspended due to the device's unavailability, there remains hope for future availability. The study did not include a control group using traditional endoscopic techniques, making it difficult to directly compare the outcomes and assess the specific benefits of the MICAS device.

## Conclusions

The MICAS device succeeded in providing stability and improved maneuverability of the endoscope. No difficulty was encountered in positioning the device or using the endoscope. Designed to shield the brain at the burr hole, the device successfully protected the site while the surgeon addressed the relevant pathology. The need for further exploration via a second burr hole can be predicted after inspection with the flexible endoscope. This safety study demonstrated reasonable risks to use of this device.
